# First-in-Human Percutaneous Cor Triatriatum Sinistrum Balloon Membranoplasty to Facilitate Percutaneous Mitral Transcatheter Edge-to-Edge Repair

**DOI:** 10.1016/j.jaccas.2024.103227

**Published:** 2025-02-26

**Authors:** Hafez Golzarian, Anna C. Kleman, Mallory Knous, Andrew H. Macke, William C. Carder, Jennifer A. Music, Amy K. Carder, Craig Imm, Nicole A. Nelson, Sandeep M. Patel

**Affiliations:** a Internal Medicine Residency Program, Mercy Health–St Rita’s Medical Center, Lima, Ohio, USA; b Structural Heart & Intervention Center, Mercy Health–St Rita’s Medical Center, Lima, Ohio, USA; c Department of Anesthesia, Mercy Health–St Rita’s Medical Center, Lima, Ohio, USA; d Department of Radiology, Mercy Health–St Rita’s Medical Center, Lima, Ohio, USA

**Keywords:** Cor triatriatum sinistrum, echocardiography, adulthood, congenital, heart failure, membranoplasty

## Abstract

**Background:**

Cor triatriatum sinistrum (CTS) is a rare congenital structural heart defect conventionally managed surgically early in life. However, at times it is discovered much later.

**Case Summary:**

We present the first in-human case of a transcatheter CTS membranoplasty with mitral transcatheter edge-to-edge repair (mTEER) in an elderly patient with CTS. Ultimately, the patient experienced reduction in mitral regurgitation (MR), translating to significant symptomatic improvement.

**Discussion:**

Although surgical resection is the standard treatment for CTS, this approach may not be suitable for high-risk elderly individuals with multiple comorbidities. To address this, we employed a novel percutaneous technique, using multiple guidewires and balloons to facilitate controlled traversal and dilation of the atrial membrane, creating adequate space for mTEER.

**Take-Home Message:**

Percutaneous membranoplasty combined with mTEER may offer a feasible and potentially preferable alternative to surgery for elderly patients with CTS who are considered high-risk surgical candidates.

## Graphical Abstract

**Figure:**
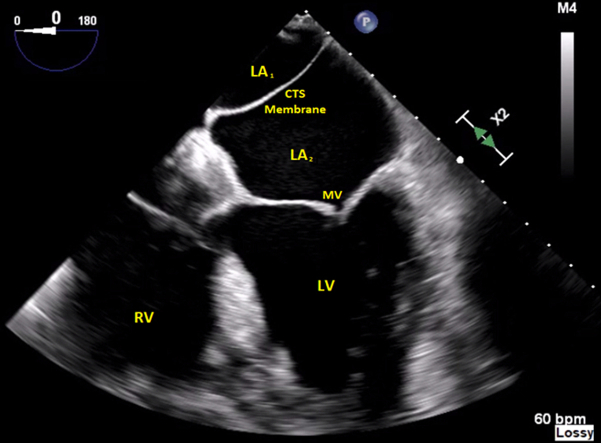

Cor triatriatum sinistrum (CTS) is a rare congenital heart defect involving a membrane that divides the left atrium into 2 chambers. This condition is often overlooked, and its association with mitral regurgitation (MR) is not well documented, especially in older patients.^1^^,^^2^ Despite advances in treatment, surgical resection remains the standard approach regardless of age. We present the first successful use of transcatheter membranoplasty and percutaneous mitral repair in an elderly patient with CTS and severe MR, suggesting this approach may be a safe and viable option for such patients.

**Learning Objectives**

• To describe in detail the procedural methodology which allowed for successful membranoplasty and mTEER in a patient with cor triatriatum sinistrum.
• To discuss standard of care for patients with symptomatic CTS and why transcatheter membranoplasty may be feasible, safe, and preferred in the elderly population.

## History of Presentation

**Figure 1:**
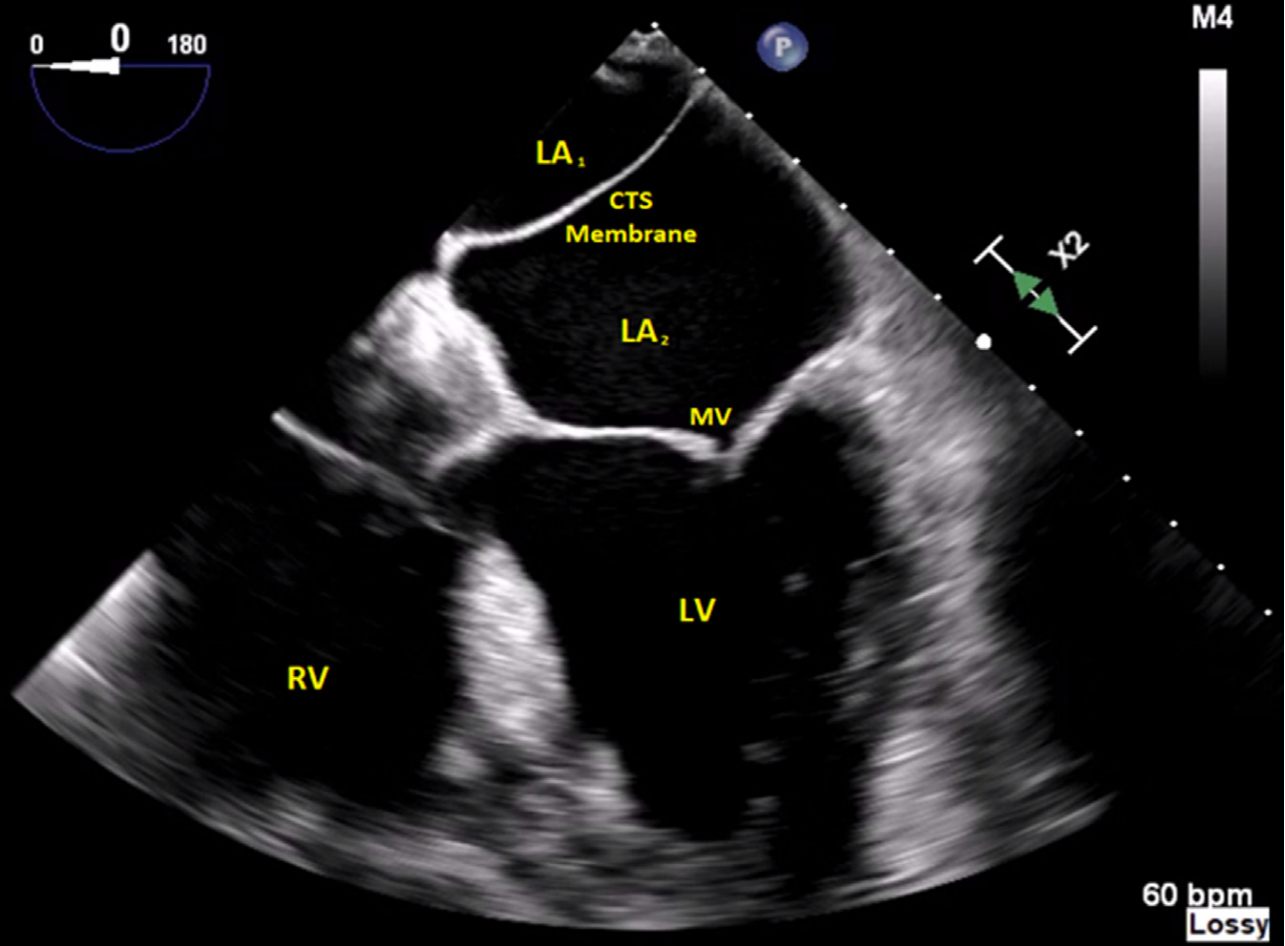
Transesophageal Echocardiography Revealing an Interatrial Membrane Consistent With Cor Triatriatum Sinistrum CTS = cor triatriatum sinistrum; LA = left atrium; LV = left ventricle; MV = mitral valve; RV = right ventricle.

A 74-year-old male with two previous cardiac bypass surgeries, hyperlipidemia, and hypertension presented with shortness of breath, palpitations, and chest pain (NYHA functional class III). Echocardiography showed severe cardiomyopathy with an ejection fraction of 25% to 30%, moderate-to-severe functional MR, and an echogenic membrane in the left atrium, consistent with CTS (Figure 1). The effective regurgitant orifice area was 0.49 cm^2^, regurgitant fraction was >50%, and regurgitant volume was 85.75 mL. Diastolic and systolic left ventricular dimensions were 5.8 cm and 5.2 cm, respectively.

Initial right and left heart catheterizations demonstrated no concerning abnormalities. The patient was subsequently managed with pharmacologic heart failure therapies, which offered temporary symptomatic improvement. His left ventricular ejection fraction did transiently improve from 25% to 30% to 45% to 50% over a 6-month period. Nevertheless, the patient's MR persisted, and his symptomatology eventually became refractory (NYHA functional class III) to ongoing medical management over the ensuing 1 to 2 years. Given the high surgical risk, a shared decision between the patient and the heart team was made to pursue percutaneous mitral transcatheter edge-to-edge repair (mTEER), despite his congenital anatomic condition.

## Preprocedural Imaging

**Figure 2:**
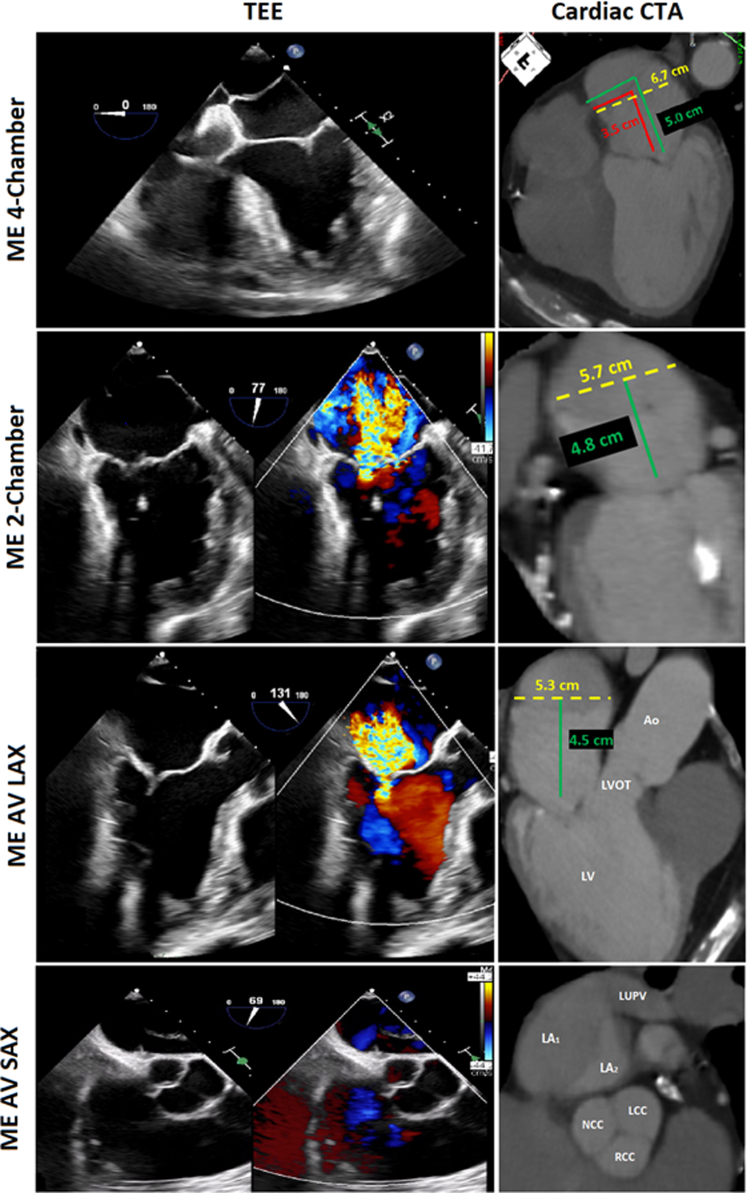
Preprocedural Cardiac CT with Coregistered TEE Images Various configurations of the left atrial membrane with coregistered TEE measurements revealing a small window for posterior puncture placed above the membrane. The membrane attaches anteriorly on the interatrial septum, connecting the left upper pulmonary vein to the anterior region of the fossa ovalis. Yellow dotted lines delineate LA width. Red line delineates the membrane height from the mitral valve. Green delineates the planned site of puncture of the interatrial septum. AV = aortic valve; CT = computed tomography; LA = left atria; LAX = long-axis; LCC = left coronary cusp; LV = left ventricle; LVOT = left ventricular outflow tract; LUPV = left upper pulmonary vein; ME = mid-esophageal; NCC = noncoronary cusp; RCC = right coronary cusp; RV = right ventricle; SAX = short-axis; TEE = transesophageal echocardiography.

Transesophageal echocardiography (TEE) and 3-dimensional analysis revealed that the membrane was complete without any fenestrations and attached to the interatrial septum 3.5 to 4.0 cm above the mitral annulus, with an area of nonfusion to the left atrial wall, allowing communication above and below. As for leaflet anatomy, functional annular dilation was noted with mildly thickened leaflets, and overriding A2 scallop with concomitant restriction of the P2 scallop were identified. Computed tomography angiography (CTA) was used to plan and coregister the TEE imaging to guide the procedure (Figure 2). This imaging suggested that we traverse 4.5 to 5.0 cm above the annulus, in the pulmonary venous portion of the left atrium, thus requiring a second puncture through the membrane's center above the region of most severe regurgitation. The membrane was surmised to be fibrous and would require balloon dilation to allow for mobility and positioning of the steerable guiding catheter. The dimensions of the left atrium were evaluated to determine the appropriate balloon sizes needed for the membranoplasty while avoiding injury to the mitral valve, atrial walls, or interatrial septum (Figure 2).

## Procedural Methodology

Using a modified Seldinger technique under fluoroscopic guidance, an 8F sheath was inserted in the right femoral vein and sequentially dilated up to a 26-F Gore DrySeal sheath (Gore Medical). Heparin was administered intravenously to maintain an activated clotting time of >250 seconds. Under TEE guidance, a posterior mid-transseptal puncture was made using an 8.5-F 45-° VersaCross sheath (Boston Scientific) and a 0.035-inch radiofrequency pigtail wire, approximately 4.5 to 5 cm above the mitral annulus. After entering the pulmonary venous left atrium, we exchanged for an Agilis medium curve sheath (Abbott Cardiovascular). Using TEE guidance in the 0°/4-chamber and 60°/2-chamber views, the Agilis sheath was flexed down toward the membrane. Once the membrane was tented with the Agilis sheath and dilator, multiple views confirmed the position above the mitral valve region for the planned mTEER. The transseptal wire was then advanced back to the tip of the Agilis sheath-dilator, and its position was verified. The wire was activated again and crossed into the left atrial appendage side of the membrane, and then advanced across the mitral valve into the left ventricle.

**Figure 3:**
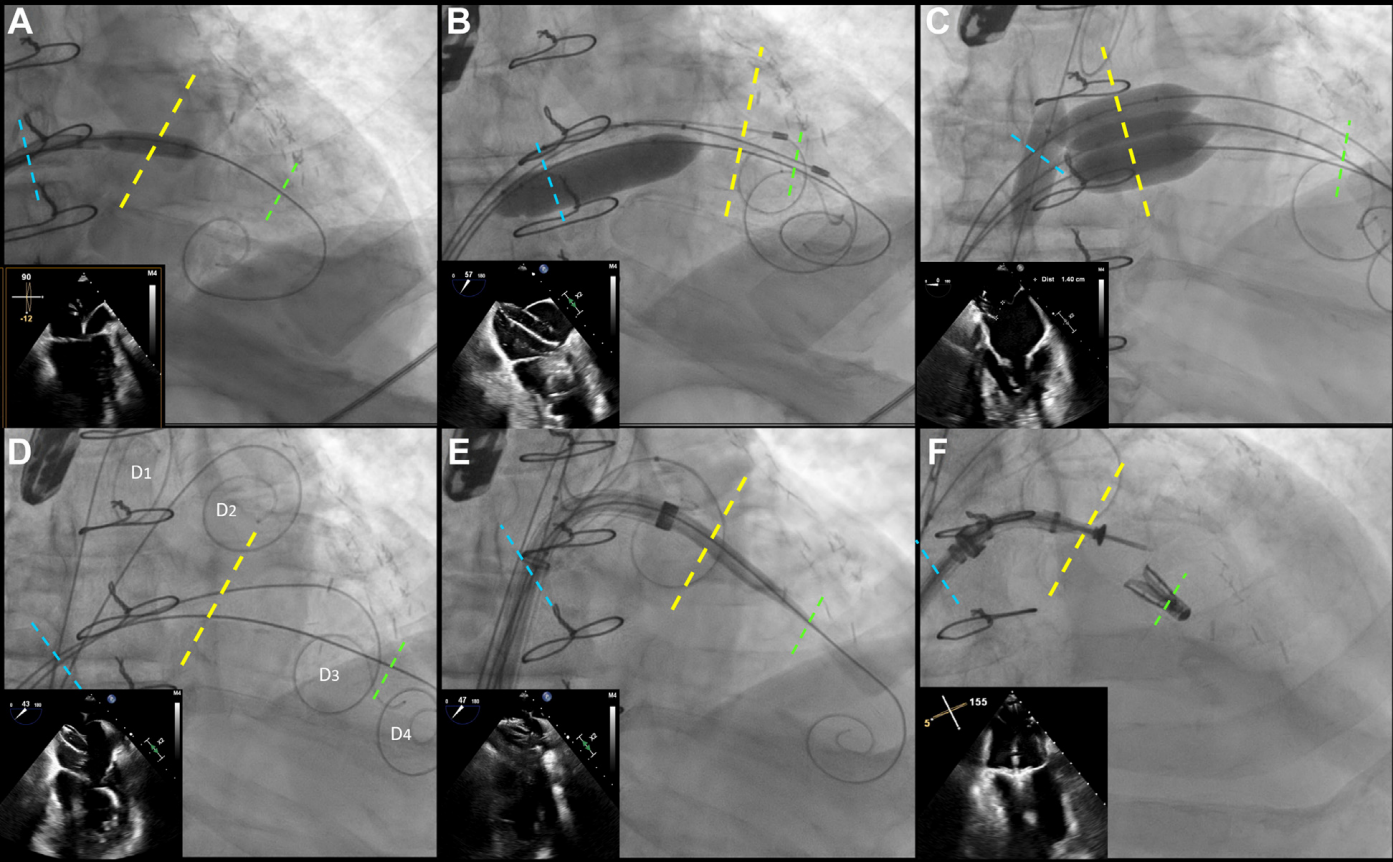
Fluoroscopic Visualizations of Procedural Steps Fluoroscopy demonstrating the various steps in traversing through the CTS membrane and successfully delivering the MitraClip. Blue dashes demarcate the interatrial septum. Yellow dashes demarcate the CTS membrane. Green dashes demarcate the mitral valve. (A) Transeptal puncture and septostomy. (B) Positioning of guidewires to pass balloons through before membranostomy. (C) Dilatations of the balloons to 6 to 10 atmospheres. (D) Postmembranoplasty positioning of Safari guidewires; Wire D_1_ positioned at the pulmonary vein, D_2_ positioned above membrane, D_3_ positioned below membrane, D_4_ positioned in left ventricle. (E) Advancement of the steerable guiding catheter. (F) Successful deployment of the MitraClip. Abbreviation as in Figure 1.

**Figure 4:**
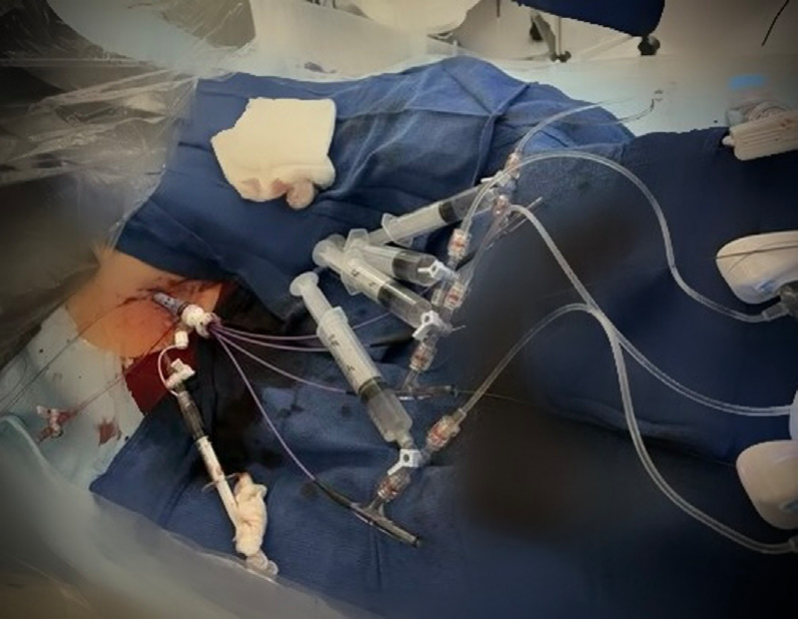
Deployment of 4 Armada Balloons Dilated at 6 to 10 atm Required to Perform Successful Membranoplasty

Using a 6 × 40 mm Armada balloon (Abbott Cardiovascular), we predilated the membrane to allow the Agilis sheath to advance completely into the left ventricle (Figure 3A), as the dilator alone could not cross the thick fibrous membrane. After inserting 2 additional Safari wires (Boston Scientific) into the left ventricle and removing the Agilis sheath (Figure 3B), our priority was to ensure safe membranoplasty, which would require using very short balloons. We initially attempted to use 2 16 × 40 mm Conquest balloons based on the atrial dimensions, but real-time echocardiography showed they were too long and risked expanding the interatrial septostomy. We then downsized to a combination of 2 14 mm and 1 12 mm × 20 mm Armada balloons, which we inserted in parallel across the CTS membrane. However, the balloons persistently gravitated back into the atrial septum, which we wanted to avoid. To resolve this, we placed a 10-mm × 40-mm blocking Armada balloon across the interatrial septostomy to prevent the other balloons from inadvertently crossing back over. We were then able successfully to inflate all 3 balloons simultaneously in 3 separate 6 to 10 atmosphere inflations to open the membrane sufficiently (Figures 3C and 4). TEE confirmed we now had a window in the membrane of at least 1.5 to 2 cm to work through.

After removing the balloons, we positioned extra-small Safari wires above and below the membrane to maintain control over the area (Figure 3D). We left a 12-mm and a 14-mm balloon in place over each of these wires to facilitate further procedures, if needed. We also kept a 0.035-inch Safari wire in the left ventricle to guide the steerable guiding catheter through the septostomy and membranostomy for the deployment of the MitraClip (Abbott Cardiovascular).

**Figure 5:**
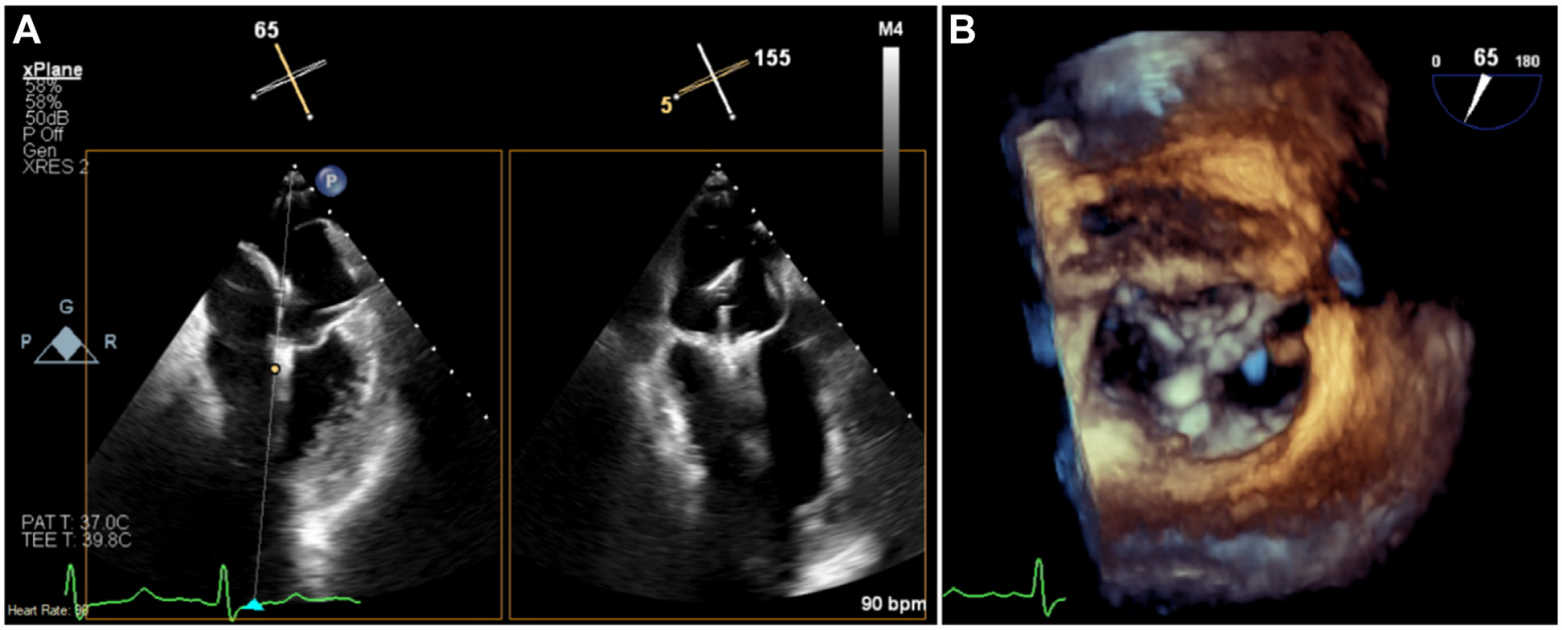
Predeployment Mitral Commissural View Successful capture of leaflets (A) followed by successful delivery of the MitraClip and visualization on 3-dimensional echocardiography (B).

We then exchanged the 26-F Gore DrySeal sheath for a standard steerable guiding catheter, which we advanced over a 0.035-inch Safari wire toward the left ventricle underneath the membrane (Figure 3E). After confirming the catheter's position beneath the membrane using TEE, we removed the wire and prepped the standard MitraClip XTW delivery system, inserting it into the steerable guiding catheter. After navigating through the cor triatrium membrane and applying posterior torque, we were able to advance the MitraClip and the fluoroscopic markers appropriately; we did need to back out the guiding catheter to allow for appropriate positioning, but at all times we ensure the tip of the guide was below the membrane. This step may necessitate multiple minor iterations and adjustments to achieve proper placement. We then advanced the clip and used medial deflection and posterior tilt to position above the most severe portion of the mitral regurgitation. We were then able to advance the clip into the left ventricle and performed grasping in standard long-axis views with the use of multiplanar reconstruction echocardiography. Ultimately, we were able to successfully deploy the MitraClip with low mean gradient of 2 to 3 mm Hg across the mitral valve and trace residual mitral regurgitation. (Figures 3F and 5). The venotomy was closed with a modified figure-of-8 suture closure.

## Follow-Up

Our patient reported symptomatic relief within hours of mTEER. His serum pro-B-type natriuretic peptide levels improved from 4,756 pg/mL (preprocedural) to 707 pg/mL within weeks. He is now 1 year postprocedure and doing well (NYHA functional class II) with only trace-to-mild MR (Video 1).

## Discussion

CTS is a rare congenital heart defect with only a few hundred cases ever reported. The severity depends on the anatomy, such as the number and size of openings in the membrane, its thickness and length, and its proximity to the pulmonary veins or mitral valve. Patients with symptomatic CTS often present with symptoms similar to mitral stenosis.^3^, ^4^, ^5^ TEE is the preferred diagnostic modality, and surgical repair is the standard definitive management.

The available literature on CTS primarily focuses on younger patients, with limited data on elderly individuals.^2^ Surgical repair has shown good long-term outcomes, but, again, the studies demonstrating such predominantly involved pediatric and adolescent populations.^5^ In our case, our patient had lived most of his life without significant CTS-related symptoms, and the initial concern was a reduction in ejection fraction and severe MR. This case highlights the need for further research and exploration of percutaneous management options for elderly patients with CTS who may not be suitable for traditional surgical interventions.

CTS is typically classified into 3 groups based on the severity of obstruction caused by the membrane.^6^ Group 1 represents complete obstruction, often requiring surgery in early childhood. Group 2 has moderate-to-severe obstruction with small fenestrations, leading to symptoms in adolescence or early adulthood, again managed surgically. Group 3 is the least symptomatic, with adequate flow through or around the membrane, either via numerous fenestrations or a large single opening. These patients may remain asymptomatic until an unrelated cardiac issue arises, which can be managed surgically or potentially with percutaneous approaches.

Identifying the etiology of mitral regurgitation is crucial, as it can affect the indication, prognosis, and procedural planning for repair. Surgery remains the standard of care for primary MR. However, in patients with high or prohibitive surgical risk and suitable anatomy, mTEER has a Class IIa recommendation for select patients with NYHA functional class III/IV. For secondary MR, guidelines suggest a Class IIa indication for mTEER in select patients with suitable anatomy who fulfill the COAPT (Cardiovascular Outcomes Assessment of the MitraClip Percutaneous Therapy) criteria. Patients who do not meet the COAPT criteria may also undergo mTEER if it improves symptoms and quality of life, although this remains a Class IIb recommendation. It is important to note that patients who meet the COAPT criteria tend to have better outcomes in terms of recurrent hospitalizations and overall mortality. In our patient, the MR was thought to be multifactorial, with degenerative, functional, and even partially ischemic components. In addition, our patient was not a surgical candidate because of 2 previous sternotomies and frailty. Therefore, mTEER remained the most favorable treatment option.^7^

We have demonstrated the feasibility of performing membranoplasty and mTEER in an elderly patient with group 3 CTS who was a poor surgical candidate. This technique can also be applied to patients with any severity of CTS, as the key is the ability to traverse the membrane while maintaining optimal positioning. Preprocedural planning and intraprocedural imaging, such as cardiac CTA/magnetic resonance imaging (MRI), and TEE, are critical for success. During the procedure, we recommend using multiple guidewires above, below, and in the left ventricle to allow for guidance and rewirability of the steerable guiding catheter. Balloon dilation catheters, such as standard peripheral balloons or an INOUE percutaneous mitral commissurotomy balloon (Toray International America Inc), can be considered, although the latter may have potential disadvantages. Regarding balloon selection, a single larger balloon was initially attempted but found to be too expansive for the patient's atrial dimensions after transeptal access. To mitigate the risk of atrial wall trauma, we instead opted for a more tailored approach, using 3 smaller balloons in parallel fashion.

We anticipate that, in the near future, percutaneous solutions will become more available for patients with cor triatriatum who are poor surgical candidates. As percutaneous methods continue to evolve, including tissue extraction and electrosurgical techniques, cor triatriatum may no longer hinder valvular repair or replacement interventions. However, additional studies are still needed to compare the safety and efficacy of these percutaneous approaches with conventional surgical interventions.

### Data Availability Statement

The data that support the findings of this study are available from the authors upon request.

## Funding Support and Author Disclosures

The authors have reported that they have no relationships relevant to the contents of this paper to disclose.

## Appendix

Video 1 Echocardiographic results pre- and post-mTEER, demonstrating successful treatment of the patient’s severe MR.
